# Design and Expression of Specific Hybrid Lantibiotics Active Against Pathogenic *Clostridium* spp.

**DOI:** 10.3389/fmicb.2019.02154

**Published:** 2019-09-24

**Authors:** Rubén Cebrián, Alicia Macia-Valero, Afif P. Jati, Oscar P. Kuipers

**Affiliations:** Department of Molecular Genetics, Groningen Biomolecular Sciences and Biotechnology Institute, Faculty of Science and Engineering, University of Groningen, Groningen, Netherlands

**Keywords:** genome mining, *Clostridium difficile*, antimicrobial susceptibility, lantibiotic design, nisin

## Abstract

*Clostridium difficile* has been reported as the most common cause of nosocomial diarrhea (antibiotic-associated diarrhea), resulting in significant morbidity and mortality in hospitalized patients. The resistance of the clostridial spores to antibiotics and their side effects on the gut microbiota are two factors related to the emergence of infection and its relapses. Lantibiotics provide an innovative alternative for cell growth inhibition due to their dual mechanism of action (membrane pore-forming and cell wall synthesis inhibition) and low resistance rate. Based on the fact that bacteriocins are usually active against bacteria closely related to the producer strains, a new dual approach combining genome mining and synthetic biology was performed, by designing new lantibiotics with high activity and specificity toward *Clostridium*. We first attempted the heterologous expression of putative lantibiotics identified following *Clostridium* genome mining. Subsequently, we designed new hybrid lantibiotics combining the start or end of the putative clostridial peptides and the start or end parts of nisin. The designed peptides were cloned and expressed using the nisin biosynthetic machinery in *Lactococcus lactis*. From the 20 initial peptides, only 1 fulfilled the requirements established in this work to be considered as a good candidate: high heterologous production level and high specificity/activity against clostridial species. The high specificity and activity observed for the peptide AMV10 makes it an interesting candidate as an alternative to traditional antibiotics in the treatment of *C. difficile* infections, avoiding side effects and protecting the normal gut microbiota.

## Introduction

The genus *Clostridium* comprises about 150 metabolically diverse species of Gram-positive, endospore-forming anaerobic bacteria that are ubiquitous in virtually all anoxic habitats where organic compounds are present, including soils, aquatic sediments, and the intestinal tracts of animals and humans (Udaondo et al., 2017). Although almost all *Clostridium* spp. are commensal strains, some species of clostridia (e.g., *C. perfringens*, *C. botulinum*, *C. tetani*, or *C. difficile*) are known to be opportunistic, toxin-producing pathogens in both animals and humans (Cassir et al., 2016; Kiu and Hall, 2018; Czepiel et al., 2019). Between the pathogenic clostridia, *C. difficile* is gaining increased attention from the research community because of its ability to escape the biocidal action of antibiotics and because of the growing number of infections especially in hospitals, where *C. difficile* is one of the most common acquired infections (Czepiel et al., 2019). In fact, *C. difficile* has been recognized as the most frequent pathogen in nosocomial diseases in Europe, causing diarrhea or pseudomembranous colitis. It has always been related to the elderly until the start of the new millennium, when numerous studies have described several outbreaks in Europe (Bauer et al., 2011), with more than 150,000 cases per year of *C. difficile* infection and a 20-fold increase of mortality. Those events have been attributed to the emergence of new and more virulent strains (Mastrantonio and Rupnik, 2018). The guidelines for treatment against *C. difficile* infections include non-antimicrobial therapies such as fecal microbiota transplantation (FMT). There has been evidence of the effectiveness of FMT (Ramai et al., 2019). In fact, in most cases, this is sufficient for full resolution of the disease (∼25% of patients) (Collins and Auchtung, 2017). Although FMT is recommended for *C. difficile* treatment, some adverse effects as nausea, abdominal pain, and FMT−related diarrhea have been observed in about 20% of the cases, and more severe adverse effects happen in about the 3% of the cases (Wang et al., 2016; Cheng et al., 2019). In terms of antimicrobial treatments, some antibiotics such as vancomycin and metronidazole are used. Nevertheless, these antibiotics do not affect spores, and for this reason, the treatments have to be administered for a prolonged time. Antibiotics also lead to the disruption of the gut microbiota because of their low specificity, allowing the pathogen to proliferate and colonize the human gut after the treatment (Mathias et al., 2019). Moreover, the misuse of antibiotics has a central role in the emergence of novel and more virulent strains, characterized by higher antibiotic resistance and toxin production (Yakob et al., 2015; Candel-Pérez et al., 2019; Fatima and Aziz, 2019). Several different strains have been reported to show a decrease in susceptibility or to be resistant to more than one antibiotic (Peng et al., 2017). Factors and mechanisms responsible for resistance include chromosomal genes, mobile genetic elements, biofilm formation and modification of antibiotic targets or metabolic pathways, among others. As a result, the development of novel specific antimicrobial compounds against *Clostridium* spp. turn out to be a necessity and a very relevant line of investigation.

Recent studies showed the potential of lantibiotics as an alternative to conventional antibiotics (van Heel et al., 2011; Hudson and Mitchell, 2018; Lewies et al., 2018). The term lantibiotic refers to lanthionine containing peptides with antimicrobial activity. They are ribosomally synthesized peptides produced mainly by Gram-positive bacteria and characterized by the presence of the atypical amino acid as dehydrobutyrine and dehydroalanine (formed after dehydration of threonine and serine residues respectively). These amino acids can react with the SH group of a cysteine forming a thioether-linked amino acid called lanthionine or a methyl-lanthionine ring, increasing the stability of the peptides (de Vos et al., 1995; van Kraaij et al., 1999; Alvarez-Sieiro et al., 2016; Repka et al., 2017). Lantibiotics are also characterized by their low resistance level of their targets. This is because most of them have multiple modes of actions: pore-forming on the cell walls causing ATP leakage or the sequestration of cell wall precursor lipid II, that inhibits the cell wall synthesis and the replication (Sahl et al., 1995; Breukink and de Kruijff, 2006; Hasper et al., 2006).

The combination of genome mining and synthetic biology approaches can be used for the identification of novel lantibiotics and then employ the modularity and the orthogonality of engineering into the design of novel powerful antimicrobial peptides (van Heel et al., 2016; Montalbán-López et al., 2017; Schmitt et al., 2019). *In silico* analysis of putative lantibiotic genes using bioinformatic tools as BAGEL4 or Anti-Smash (Weber et al., 2015; van Heel et al., 2018) provides an accurate prediction of putative novel lantibiotics. BAGEL4 software can analyze DNA sequences by two different approaches. First, an indirect approach which is the context of bacteriocin- or RiPP gene-based mining and then a direct approach, which is structural gene-based mining directly via Glimmer software for finding genes in microbes (van Heel et al., 2018, 4). These approaches improve the success rate by reducing the false positive probability and minimize manual evaluation of results. Also, anti-SMASH is an application that predicts putative bacteriocin genes as well as their biochemical properties, and further details including gene cluster description, annotation, and genomic loci for the biosynthetic pathway (Weber et al., 2015). Combination of BAGEL4 and anti-SMASH for genome mining gives accurate information for identification of unknown lantibiotic genes in various organisms. Afterward, the new DNA sequence encoding the putative lanthipeptide gene can be fused to the nisin leader sequence and expressed heterologously in *Lactococcus lactis* (Montalbán-López et al., 2017). Alternatively, the design of hybrid lantibiotics by a combination of known ones, with enhanced antimicrobial activity has also been described as a potent tool for the identification of new drugs (Schmitt et al., 2019).

With some exceptions, bacteriocin/lantibiotics are usually active against strains closely related to the producer one (Yang et al., 2014; Todorov et al., 2019). The use of new peptides with high specific activity against *Clostridium* spp. and with low or no activity against other bacteria would provide a good strategy in the treatment of *C. difficile* infections, minimizing or limiting one of the most unwanted side effects of traditional antibiotics after prolonged treatments, i.e., gut microbiota modification. This alteration is related to the recurrence of the infection and also to other pathologies such as diarrhea (Nelson, 2007; Song et al., 2008).

This study is focused on the identification, design, and production of new lantibiotics with enhanced activity against *C. difficile*. These new peptides must fulfill three requirements: high heterologous production using the nisin biosynthetic machinery, and high specificity and activity toward *C. difficile*.

## Materials and Methods

### Microorganisms, Plasmids, and Growth Conditions

The strains used in this work and the plasmid designs are listed in Table 1. *Escherichia coli* strains were grown in LB medium at 37°C and shaking. *Lactococcus* and *Lactobacillus* strains were grown in M17 + 0.5% of glucose (GM17) at 30°C without shaking. *Bacillus*, *Enterococcus*, *Listeria*, *Staphylococcus*, and *Streptococcus* strains were grown as described above but at 37°C. *Clostridium* strains were grown in Reinforced Clostridium Medium (RCM) at 37°C in anaerobiosis in a Coy Anaerobic Chamber. For solid media, agar at 1.2% was added. For Amp^R^ resistant plasmids selection in *E. coli*, 100 μg/mL of ampicillin were added, while 5 or 10 μg/mL of chloramphenicol/erythromycin were added for *L. lactis* selection.

**TABLE 1:** Strains and plasmids used in this work.

**Strain**	**Characteristic/purpose**	**References**
*Escherichia coli* TOP-10	*mcrA*, Δ(*mrr-hsdRMS-mcrBC*), *Phi80lacZ*(*del*)*M15*, Δ*lacX74*, *deoR*, *recA1*, *araD139*, Δ(*ara-leu*)*7697*, *galU*, *galK*, *rpsL*(*SmR*), *endA1*, *nupG*	Thermo Fisher Scientific
pUC57-Clos_x_	Amp^R^, synthetic gene design	This work
pUC57-AMV_x_	Amp^R^, synthetic gene design	This work
*Lactococcus lactis* NZ9000	*pep*N*:nisRK*	de Ruyter et al., 1996
pIL253 pNZe-NisP8H	Ery^R^, Cm^R^, NisP producer strain	Montalbán-López et al., 2018
pTLR-BTC	Ery^R^, *pepN:nisRK*, *nisBTC* genes cloned from pIL3-BTC into pTLR plasmid	Lab collection
pTLR-BTC pNZ8048	Ery^R^, Cm^R^, *pepN:nisRK*, *nis*BTC	This work
pIL3-BTC pNZ8048-NisA	Ery^R^, Cm^R^, NisA producer strain	van Heel et al., 2013
pTLR-BTC pNZ8048-Clos2	Ery^R^, Cm^R^, synthetic gene *clos2* cloned fused to nisin leader under P_nis_ promoter in pNZ8048-NisA	This work
pTLR-BTC pNZ8048-Clos4	Ery^R^, Cm^R^, synthetic gene *clos4* cloned fused to nisin leader under P_nis_ promoter in pNZ8048-NisA	This work
pTLR-BTC pNZ8048-Clos5	Ery^R^, Cm^R^, synthetic gene *clos5* cloned fused to nisin leader under P_nis_ promoter in pNZ8048-NisA	This work
pTLR-BTC pNZ8048-Clos12	Ery^R^, Cm^R^, synthetic gene *clos12* cloned fused to nisin leader under P_nis_ promoter in pNZ8048-NisA	This work
pTLR-BTC pNZ8048-Clos14	Ery^R^, Cm^R^, synthetic gene *clos14* cloned fused to nisin leader under P_nis_ promoter in pNZ8048-NisA	This work
pTLR-BTC pNZ8048-Clos15	Ery^R^, Cm^R^, synthetic gene *clos15* cloned fused to nisin leader under P_nis_ promoter in pNZ8048-NisA	This work
pTLR-BTC pNZ8048-Clos16	Ery^R^, Cm^R^, synthetic gene *clos16* cloned fused to nisin leader under P_nis_ promoter in pNZ8048-NisA	This work
pTLR-BTC pNZ8048-Clos17	Ery^R^, Cm^R^, synthetic gene *clos17* cloned fused to nisin leader under P_nis_ promoter in pNZ8048-NisA	This work
pTLR-BTC pNZ8048-Clos22	Ery^R^, Cm^R^, synthetic gene *clos22* cloned fused to nisin leader under P_nis_ promoter in pNZ8048-NisA	This work
pTLR-BTC pNZ8048-Clos24	Ery^R^, Cm^R^, synthetic gene *clos24* cloned fused to nisin leader under P_nis_ promoter in pNZ8048-NisA	This work
pTLR-BTC pNZ8048-AMV1	Ery^R^, Cm^R^, synthetic gene *AMV1* cloned fused to nisin leader under P_nis_ promoter in pNZ8048-NisA	This work
pTLR-BTC pNZ8048-AMV2	Ery^R^, Cm^R^, synthetic gene *AMV2* cloned fused to nisin leader under P_nis_ promoter in pNZ8048-NisA	This work
pTLR-BTC pNZ8048-AMV3	Ery^R^, Cm^R^, synthetic gene *AMV3* cloned fused to nisin leader under P_nis_ promoter in pNZ8048-NisA	This work
pTLR-BTC pNZ8048-AMV4	Ery^R^, Cm^R^, synthetic gene *AMV4* cloned fused to nisin leader under P_nis_ promoter in pNZ8048-NisA	This work
pTLR-BTC pNZ8048-AMV5	Ery^R^, Cm^R^, synthetic gene *AMV5* cloned fused to nisin leader under P_nis_ promoter in pNZ8048-NisA	This work
pTLR-BTC pNZ8048-AMV6	Ery^R^, Cm^R^, synthetic gene *AMV6* cloned fused to nisin leader under P_nis_ promoter in pNZ8048-NisA	This work
pTLR-BTC pNZ8048-AMV7	Ery^R^, Cm^R^, synthetic gene *AMV7* cloned fused to nisin leader under P_nis_ promoter in pNZ8048-NisA	This work
pTLR-BTC pNZ8048-AMV8	Ery^R^, Cm^R^, synthetic gene *AMV8* cloned fused to nisin leader under P_nis_ promoter in pNZ8048-NisA	This work
pTLR-BTC pNZ8048-AMV9	Ery^R^, Cm^R^, synthetic gene *AMV9* cloned fused to nisin leader under P_nis_ promoter in pNZ8048-NisA	This work
pTLR-BTC pNZ8048-AMV10	Ery^R^, Cm^R^, synthetic gene *AMV10* cloned fused to nisin leader under P_nis_ promoter in pNZ8048-NisA	This work
*Clostridium beijerinckii* NIZO B504	Indicator strain	Lab collection
*C. botulinum* CECT551	Indicator strain	CECT
*C. difficile* CECT531	Indicator strain	CECT
*C. ihumii* AP5	Indicator strain	Merhej et al., 2015
*C. sporogenes* C22/10	Indicator strain	Aktypis et al., 1998
*C. tyrobutyricum* NIZO B574	Indicator strain	Lab collection
*Bacillus cereus* ATCC10987	Indicator strain	ATCC
*Enterococcus faecalis* LMG8222	Indicator strain	LMG
*E. faecium* LMG16003	Indicator strain	LMG
*Listeria monocytogenes* LMG10470	Indicator strain	LMG
*Lactobacillus plantarum* WCFS1	Indicator strain	Kleerebezem et al., 2003
*Lactococcus lactis* MG1363	Indicator strain	Wegmann et al., 2007
*Staphylococcus aureus* LMG8224	Indicator strain	LMG
*Streptococcus salivarius* HSISS3	Indicator strain	Van den Bogert et al., 2013

*ATCC, American Type Culture Collection; CECT, Spanish Type Culture Collection; LMG, Belgian Co-ordinated Collections of Microorganism.*

### Genome Mining of Various *Clostridium* Strains and Synthetic Gene Design

Identification of novel putative lantibiotic genes was performed using BAGEL4 (van Heel et al., 2018) and antiSMASH (Weber et al., 2015). 563 genomes from *Clostridium* spp. strains and 43 of the recently separated *Paeniclostridium sordelii* genus (Sasi Jyothsna et al., 2016) deposited on the NCBI database were mined. Novelty, completeness of the gene cluster and the presence of lantibiotics-related protein domains, as the NisC interaction domain FxLx in the putative leader (van der Meer et al., 1994; Abts et al., 2013; Plat et al., 2013), were considered in the selection of the putative lantibiotic genes. Twenty synthetic genes (GeneScript) were designed by optimizing the codon usage to *L. lactis* using the Jcat program (Grote et al., 2005). The first 10, with the selected-putative-lantibiotic genes identified after genome mining (pUC-Clos_x_) and the second 10 by the combination of the first or last rings of the putative lantibiotics with the first or last rings of nisin (pUC57-AMV_x_). To simplify the cloning, the genes were designed with 5′ and 3′ tags: TCGAGTTCAAAAAAAGATTCAGGTGCTAGC-gene-TAACTTTGAACCAAAATTAGAAAACCC in the case of the Clos_x_ genes and AMV_x_ hybrids with the last part of nisin, and ATTACAAGTATAAGCTTATGTACACCCGGGTGT-gene-TAACTTTGAACCAAAATTAGAAAACCC in the case of AMV_x_ hybrids with the first part of nisin. The plasmids with the designed genes were transformed into chemo-competent cells of *E. coli* TOP-10 (Green and Rogers, 2013) and then cloned into *L. lactis.*

### Molecular Cloning

The designed plasmids pUC57-Clos_x_ were isolated, and each gene was amplified with specific primers designed for USER ligation (Geu-Flores et al., 2007), Clos-USER-fw and Pep-USER-rv, and cloned into pNZ8048-NisA (van Heel et al., 2013) fused to nisin leader (instead nisin) and under P*nis* control. The backbone of pNZ8048-NisA was amplified using the primers Leader-USER-rv and pNZ-USER-fw. The same amounts of fragment and backbone were mixed and ligated using USER (NEB) enzyme according to the suppliers. After that, the ligation was dialyzed against ultra-pure water and transformed into electrocompetent cells of *L. lactis* NZ9000 pTLR-BTC (Holo and Nes, 1995), where the nisin processing enzymes (*nis*BC) and the transporter (*nis*T) were present. In the case of the other synthetic genes (pUC57-AMV_x_), the same procedure was performed with some differences regarding the primers used. For the hybrid peptides with the first part of nisin fused to the last part of the putative lantibiotics, the genes were amplified using Pep-USER-fw and Pep-USER-rv primers, while the primers Rab-USER-rv and pNZ-USER-fw were used for the backbone. For those hybrid peptides in which the last rings of nisin were fused to the first rings of the putative lantibiotics, Clos-USER-fw and Pep-USER-rv were used for the amplification of the genes, while pNZery-USER-fw and Leader-USER-rv were used for the backbone. The primer sequences and the PCR conditions are listed in Table 2. Finally, the different plasmids pNZ-Clos_x_ or pNZ-AMV_x_ were isolated, and the correct sequences of the peptides were confirmed by sequencing (Macrogen Europe, Amsterdam, Netherlands).

**TABLE 2:** Primers and PCR conditions used in this work.

**Name**	**Sequence**	**PCR conditions**
Pep-USER-fw	AGTATAAGCT*U*ATGTACA CCCGGGTGT	1× 95°C 3 min, 30× (95°C 30 s, 55°C 30 s, 68°C 30 s), 1× 68°C 3 min
Pep-USER-rv	ACCGCATGCT*U*CTCGAGGGTTT TCTAATTTTGGTTCAAAG	
Clos-USER-fw	ATCTTGTTTCAG*U*TTCAAAAAAA GATTCAGGTGCTAGCCCACGT	

pNZ-USER-fw	AAGCATGCGG*U*CTTTGAACCA AAATTAGAAAACCAAGGCTTG	1× 95°C 3 min, 30× (95°C 30 s, 55°C 30 s, 68°C 5 min), 1× 68°C 6 min
Leader-USER-rv	ACTGAAACAAGA*U*CAAGATT AAAATCTTTTGTTGAC	
Rab-USER-rv	AAGCTTATAC*U*TGTAATGCGT GGTGATGCACCTGAATC	

pNZ-Cm-fw	CATGCAGGATTGTTTATGAA CTCTATTCAGGAATTGTCAG	1× 95°C 3 min, 30× (95°C 30 s, 55°C 30 s, 68°C 1 min), 1× 68°C 3 min
pNZ-*Sph*I-rv	TCGCCGCATGCTATCAA TCAAAGCAACACGTGC	

*In italic and underlined the uracil nucleotide required for USER ligation.*

### Peptide Expression, Purification, Quantification, and Characterization

Initially, TCA precipitation (Sambrook et al., 1990) was performed in order to identify the peptides with better expression. Briefly, 50 mL of minimal expression medium (MEM) (Rink et al., 2005) was inoculated at 2% from an overnight culture of the producer strains grown in GM17 with the corresponding antibiotics. The cells were incubated at 30°C until the OD_600__nm_ reached 0.4–0.6, and at this point, nisin at 10 ng/mL was added to induce the expression. The culture was left in the same conditions overnight. The cells were removed and TCA at 10% (final concentration) was added to the supernatants, that were placed on ice for 2 h. Finally, the peptides were collected by centrifuging at 10,000 rpm for 1 h at 4°C, washed with cold acetone and resuspended in 0.5 mL of 0.05% acetic acid. The peptides were analyzed by Matrix-Assisted Laser Desorption/Ionization with a Time-Of-Flight detector (MALDI-ToF) and those peptides detected were selected for a large-scale purification using 1 L of MEM medium.

After the induction and incubation, the peptides were purified as described by Cebrián et al. (2012). Briefly, the culture pH was raised to 6 and mixed with Sephadex CM-25 (Sigma–Aldrich) (previously swollen overnight in distilled water at 4°C) at 1:10 (vol:vol). The mixture was shaken for 1 h, and then the CM-25 was decanted and placed into a chromatographic column. The CM-25 was washed with 1 L of distilled water, and the peptides were eluted with 800 mL NaCl 2 M distributed in 50 mL fractions. The active fractions were applied on reverse-phase C18 chromatography for a second purification step. Isopropanol:acetonitrile (2:1) 0.1% trifluoroacetic acid (TFA) was used as organic phase (solvent B) while 0.1% of TFA in distillated water was used as aqueous phase (solvent A). The peptides were eluted from the C18 column with 10 mL of different percentages of solvent B (10, 20, 30, 40, 50, 60, and 80%). Finally, the fractions were lyophilized, and the peptides resuspended in 1 mL of solvent A.

To obtain highly pure peptides, the previous active fractions were purified by reverse-phase C4 HPLC. For the purification, the Jupiter 5 μ C4 300A column (Phenomenex) was equilibrated in 5% of solvent B, and then a linear gradient 5–60% of solvent B was applied to elute the peptides. The *L. lactis* NZ9000 pIL253 pNZe-NisP8H strain was used as a sensitive strain in the different purification steps.

Active fractions were lyophilized, resuspended in solvent A, and quantified using a Quantus^TM^ fluorometer (Promega) and the Qubit^TM^ Protein Assay Kit (Thermo Scientific) according to the suppliers. Briefly, the samples were diluted 100-fold in 1× NanoOrange reagent working solution (1× NanoOrange protein quantitation diluent + 500-fold diluted NanoOrange protein quantification reagent), and the samples were incubated in the dark for 10 min at 93°C and then at room temperature for 20 min. After that, the fluorescence of the samples was measured in the Quantus^TM^ fluorometer (Promega). Nisin at known concertation was used as a reference.

For the nisin leader cleavage, NisP was purified from the supernatant of 1 L of *L. lactis* NZ9000 pIL253 pNZe-NisP8H [a soluble NisP producer strain (Montalbán-López et al., 2018)] in MEM medium after a nisin induction (10 ng/mL) using a Histrap^TM^ excel column (GE Healthcare) according to the suppliers. NisP was eluted with 20 mL of elution buffer (50 mM of NaH_2_PO_4_, 0.5 M NaCl, 200 mM imidazole, 20% of glycerol, pH 8) aliquoted and stored at −80°C. The ratio prenisin:NisP was optimized in 1:0.005 (prenisin 10% TCA precipitated supernatant:purified NisP) after 1 h at 37°C. The leader cleavage efficiency of the peptides was analyzed. For this, the purified peptides were mixed with NisP in the previous conditions, and the leader cleavage monitored each 1 h during 3 h using MALDI-ToF.

Finally, the dehydration level of the peptides was determined after the cleavage by MALDI-ToF according to the methodology described by van Heel et al. (2013). The average expected masses were calculated using the ProtParm program (Wilkins et al., 1999) and the percentage of each dehydration peak was calculated approximately as an average of the intensity of each peak after several MALDI-ToF analyses. The best peptides were analyzed for the ring formation using *N*-ethylmaleimide (NEM) reaction according to Yang and van der Donk (2015) methodology.

### Antimicrobial Test

Minimal inhibitory concentration (MIC) measurements using a 96-well plate microdilution method and spot overlay assays were performed as systems for antimicrobial testing. Clinical and Laboratory Standards Institute (CLSI) indications were followed as far as possible. Mueller Hinton Agar (Difco, Thermo scientific) was used in the spot overlay screening test for the indicator strains (Hockett and Baltrus, 2017). In this case, NisP and the peptides were mixed as above and spotted (5 μl) on the indicator strains. For the MIC test, NisP was added into the liquid medium (RCM) in a relation 1:0.005 and then, serial dilutions of the peptides (from 32 to 0.003 μg/mL) were performed by triplicate. Finally, the indicator strains were added at 10^5^ CFU/mL. All assays with *Clostridium* were performed in a Coy Anaerobic Chamber.

## Results

### Lantibiotics Genome Mining of *Clostridium* spp.

In general, *Clostridium* spp. are organisms relatively difficult to grow, and require complex media as well as anaerobiosis. The identification of antimicrobial peptides in this genus is usually hard, and their production at high levels is challenging. In order to identify new lantibiotics specific and active against pathogenic *Clostridium* strains as *C. difficile*, two bioinformatics programs were used. Firstly, high-throughput screening of genomic data was accomplished using AntiSMASH (Weber et al., 2015) and then small putative lantibiotic ORFs were identified using BAGEL4 (van Heel et al., 2018). 563 genomes belonging to 110 *Clostridium* species, as well as 43 strains of *P. sordelii* that had been entirely sequenced and stored in Genebank NCBI, were mined.

All genomes were uploaded on the AntiSMASH program for the first selection of possible lantibiotic sequences identification. From the 606 initial genomes, only 17 were identified harboring putative lantibiotics sequences. They were downloaded and analyzed with BAGEL4. Finally, 54 putative lantibiotic genes were detected (Supplementary Table S1). Among all putative lantibiotic detected, 10 genes were selected for heterologous expression in *L. lactis* NZ9000 pTLR-BTC.

### Putative Lantibiotics Selection, Expression, and Characterization From *Clostridium* spp.

#### Lantibiotics Selection

**FIGURE 1:**
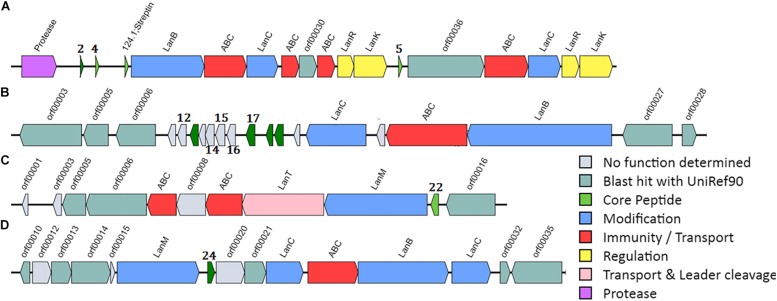
Cluster organization of the selected putative lantibiotics. **(A)** *C. beijerinckii* HUN142, **(B)** *C. ihumii* AP5, **(C)** *C. perfringens* D JGS1721, and **(D)** *Clostridium* sp. BR31.

Based on novelty, the presence of all modification enzymes in the cluster (Figure 1) and the presence of some lantibiotic-related domains, 10 putative lantibiotic genes (Table 3) were selected, synthesized, and cloned, fused to the nisin leader under the P_nis_ promoter in pNZ-8048. The designed plasmids were transformed into *L. lactis* NZ9000 cells pTLR-BTC, which provides the rest of the genes necessary for their biosynthesis. Leader sequences were assigned manually considering the known leader cutting site for other lantibiotics (presence of P in the cleavage area, GG motifs, similar cutting site to known lantibiotics), the distance from the FxLx box (Abts et al., 2013), or the position of the first C (Table 3). In order to ensure the novelty of the selected peptides, a BLASTp analysis was performed for each one.

**TABLE 3:** Putative lantibiotic sequences selected for *L. lactis* heterologous expression.

**Name**	**Strain**	**Putative leader**	**Putative core peptide**	**S + T**
Clos2	*C. beijerinckii* HUN142	VGKLDD**FDLD**VKVKINSKKGIKPS	YLSLTPKCTSLCPTNVFVCISKRCK	6
Clos4		MGKLDD**FDLD**VKVKATPKGGVKPS	ITSRILCTSSCYTQFIQCHDRV	6
Clos5		MGKLDN**FDLD**VKIKKDEKRGVKPS	VTSYSACTPGCATSLFRTCLTRSCKGC	9
Clos12	*C. ihumii* AP5	MPNYKE**FDLD**IRNEKNNLKSMNSKKRSD**GG**	TCYYSCGCKTNEGNSCGKVCFTDTIVCGTDFDGR	7
Clos14		MPNYKD**FDLD**IQNIKMNKINDKRRYPI	SDKRDDMSMCVCKKTDVCKTHETDSCNNGLCFESGKCTWV	8
Clos15		MPNYKD**FDLD**IQNSKLGVDSSRKVLPP	TFSYEYDKLSECRCRPKTQTCATHCSCATYCNGSCNQHTDCAL	10
Clos16		MPNYKE**FDLD**IRNSKNGINMYGPSAVIVP	ATDGGGKKTVCGRTCNGSACNPNSCQTRCIKPAD	6
Clos17		MPNYKD**FDLD**IQNNKSSVNSIKTTTMPP	TFSYEYDQYSECVCKPKTRNSCVTYCNGSCNQHTDCTL	9
Clos22	*C. perfringens* D JGS1721	MMKQLDKKSKTGIYVQVASDKELELLV**GG**	AGAGFIKTLTKDCPEVVSQVCGSFFGWVSACKNC	5
Clos24	*Clostridium* sp. BR31	MDD**FDLD**LRKIAENGNSANALSASDMITSEIISK	VTETITRTFKGQCVSVETPTTGMTSACCKKGGTDVEPQCVP	11

*In bold, domain related to the peptide processing. S and T positions are indicated in green while C positions are in red.*

In the case of the putative lantibiotics from *C. beijerinckii* HUN142 (Clos2, Clos4, and Clos5 in this study), the complete set of biosynthetic genes including putative modification enzymes, ABC transporters, regulation, immunity, and protease protein was identified in the cluster, which shows similarity to the class I lantibiotic biosynthetic genes cluster (Figure 1A). Interestingly, this cluster appears to be duplicated. One part could be related to Clos2 and Clos4 production and the other one with Clos5 production. Based on BLASTp homology (NCBI) analysis, these peptides belong to the gallidermin/nisin family, but low level of homology and/or similarity was observed except for Clos5. In this case, the putative lipid II binding domain region (CTPGCA) is the same as for gallidermin or nisin. Another putative antimicrobial peptide corresponding to streptin but with two modifications (G2N and M6A) was also identified in this cluster.

*Clostridium ihumii* AP5 is a new species of *Clostridium* isolated from a French Caucasian female with anorexia nervosa (Merhej et al., 2015). Surprisingly, 11 putative lantibiotic genes were identified after the mining in this strain and only one *lan*BTC putative system (Figure 1B). In their sequences, they are quite diverse, limiting the similarities to the first 15 aa of the leader peptide (Supplementary Table S1). After a BLASTp analysis of these peptides, no homologies were found, and no other lantibiotic has been described with similar characteristics. For this reason, 5 of them (Clos12, Clos14, Clos15, Clos16, and Clos17) were selected for their heterologous expression.

One single component lantibiotic from Class II lanthipeptides was found in *C. perfringens* D JGS1721 coded (Figure 1C) as Clos22 and identified as type A(II) lantibiotic which is known to have two domains, a linear N-terminal region and a globular C-terminal region. These types of lantibiotics show a slightly different mode of action than the type A(I) lantibiotics since they only can inhibit cell wall synthesis by the interaction with the lipid II (no pore-forming activity). After a BLASTp analysis, a 52% of identity with the predicted lantibiotic columbicin A was observed.

Taken from the newest study about *Clostridium* sp. BR31, this species was previously known as novel species in the genus of *Clostridium* as was still listed in *Clostridium* genome databases when this study started, but recently it had a new order in taxonomy and was declared as a new genus in *Clostridium* cluster XIVa, in the family Lachnospiraceae. This species now is designated as *Merdimonas faecis gen. nov.*, sp. nov (Seo et al., 2017). An a-typical cluster structure was observed for this strain, with two *lan*C genes separated by *lan*T and *lan*B, as well as the presence of the *lan*M gene upstream of the structural gen (Figure 1D). The unusual distribution of the cysteines in the peptide makes it difficult to place it inside the lantibiotics groups. No homologies were found with other lantibiotics after the BLASTp analysis.

#### Lantibiotics Expression and Characterization

**FIGURE 2:**
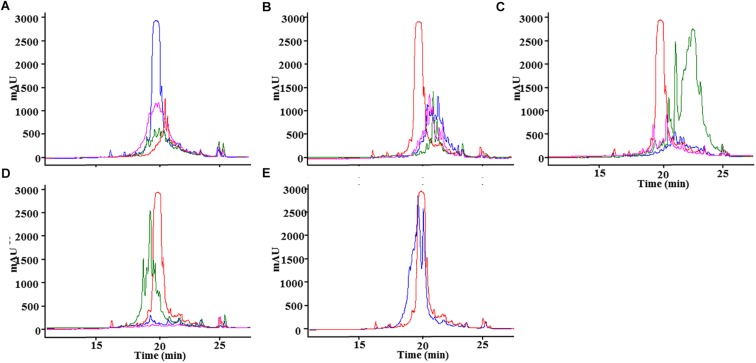
RP-HPLC chromatogram for: **(A)** nisin (blue), Clos2 (red), Clos4 (green), and Clos5 (pink) peptides. **(B)** Nisin (red), AMV1 (green), AMV2 (pink), AMV3 (blue). **(C)** Nisin (red), AMV4 (blue), AMV5 (green), AMV6 (pink). **(D)** Nisin (red), AMV7 (green), AMV8 (blue), AMV9 (pink). **(E)** Nisin (red), AMV10 (blue).

As a first approximation, 10% TCA precipitation was performed, and the samples were analyzed by MALDI-ToF. Unfortunately, from the 10 peptides, only Clos2, Clos4, and Clos5 were observed. So, these strains were selected for a larger purification using 1 L of MEM medium, CM-25, C18, and RP-HPLC (Figure 2A).

**FIGURE 3:**
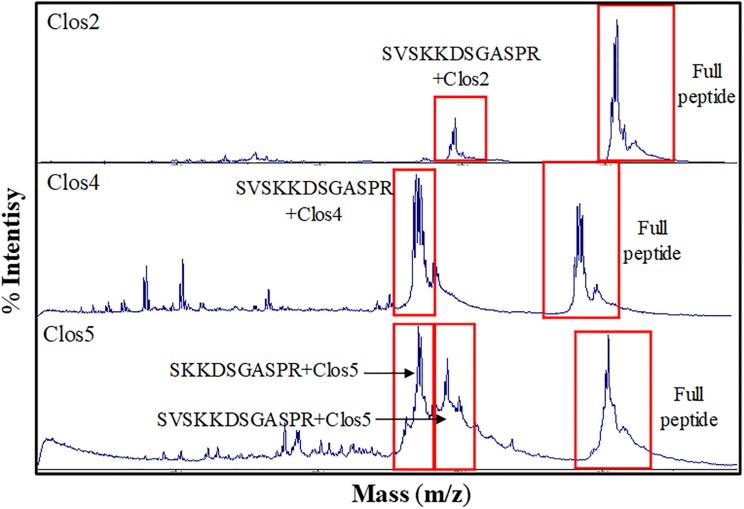
MALDI-ToF chromatogram for Clos_x_ peptides indicating different degradation levels.

The production levels obtained for the peptides in comparison with nisin were not high, being the lowest for Clos4. Many peaks were also observed indicating different levels of dehydration (hetero-dehydration) (Figure 2A). The peptides were analyzed by MALDI-ToF, and a strong hetero-dehydration, as well as degradation of the peptides, was observed, specifically in the case of Clos4 and Clos5. According to the observed masses, N-terminal degradation of the peptide leader (MSTKDFNLDLV and/or MSTKDFNLDLVSV degradation) could be related to this (Figure 3).

**FIGURE 4:**
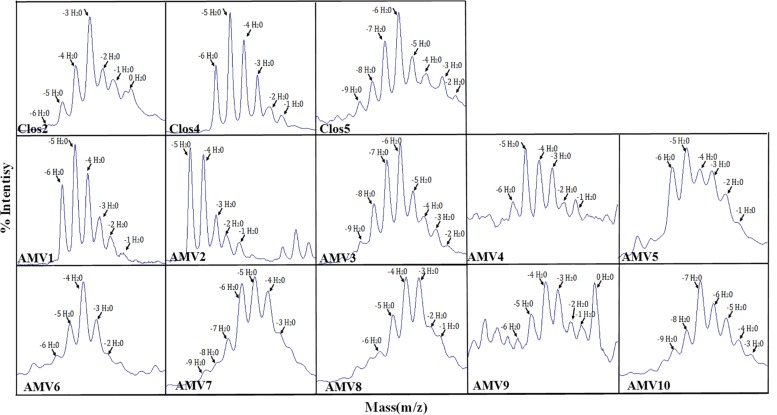
MALDI-Tof of the designed peptides after the leader cleavage, indicating the dehydration level.

Finally, in order to establish the dehydration levels for each peptide and because of the high ratio of N-terminal degradation, the peptides were digested with NisP. Firstly, the efficiency of the cleavage was analyzed monitoring the leader release during 3 h. In the case of these peptides, almost all the peptides were cleaved after this time. However, the efficiency was much lower than for nisin during the first and second hour (Supplementary Figure S1). The percentage of each dehydration level was approximately calculated considering the intensity of each peak after MALDI-ToF (Figure 4 and Table 4) (the results were similar for different MALDI-ToF analyzed samples). In general, low dehydration levels were observed. In the case of Clos2, 11.4% of the observed peptide was non dehydrated, while 29.9% were with −3 H_2_O and only the 2.4% of the peptide was fully dehydrated (−6 H_2_O) (Table 4). In the case of Clos4, it was the best dehydrated, no full dehydrated peptides (−6 H_2_O) were observed, and 21, 35, and 26.5% of dehydration was observed for −5, −4, and −3 H_2_O, respectively (Table 3). A 5.8% of the peptide was no dehydrated. Finally, for Clos5, the 6.1% of the peptide was fully dehydrated (−9 H_2_O), but the higher dehydration range was −6 H_2_O (22.7%) (Table 4).

**TABLE 4:** Molecular weight for the different AMV peptides and abundance.

**Dehy**	**Clos2**		**Clos4**		**Clos5**		**AMV1**		**AMV2**		**AMV3**		**AMV4**	
							
	**MW**	**%**	**MW**	**%**	**MW**	**%**	**MW**	**%**	**MW**	**%**	**MW**	**%**	**MW**	**%**
0	2805.4	11.4	2575.0	5.8	2817.3	–	2571.1	–	2485.9	–	2823.4	–	3021.5	–
1	2787.4	14.1	2557.0	8.3	2799.3	–	2553.1	2.67	2467.9	6.3	2805.4	–	3003.5	17.3
2	2769.4	16.7	2539.0	17.1	2781.3	7.3	2535.1	6.41	2449.9	9.0	2787.4	4.5	2985.5	17.0
3	2751.4	29.9	2521.0	26.6	2763.3	10.8	2517.1	11.63	2431.9	15.0	2769.4	8.1	2967.5	25.1
4	2733.4	17.4	2503.0	35.3	2745.3	11.3	2499.1	21.38	2413.9	33.7	2751.4	11.0	2949.5	27.9
5	2715.4	8.1	2485.0	20.9	2727.3	14.6	2481.1	30.87	2395.9	36.0	2733.4	17.0	2931.5	30.1
6	2697.4	2.4	2467.0	–	2709.3	22.7	2463.1	27.03			2715.4	27.9	2913.5	17.0
7					2691.3	17.4					2697.4	24.8	2895.5	–
8					2673.3	9.8					2679.4	14.0		
9					2655.3	6.1					2661.4	5.3		

**Dehy**	**AMV5**		**AMV6**		**AMV7**		**AMV8**		**AMV9**		**AMV10**			
						
	**MW**	**%**	**MW**	**%**	**MW**	**%**	**MW**	**%**	**MW**	**%**	**MW**	**%**		

0	3186.8	–	3406.9	–	3732.5	–	3713.5	–	3808.5	18.8	3587.3	–		
1	3168.8	4.2	3388.9	–	3714.5	–	3695.5	8.1	3790.5	11.5	3569.3	–		
2	3150.8	11.2	3370.9	10.1	3696.5	–	3677.5	12.0	3772.5	11.9	3551.3	–		
3	3132.8	19.5	3352.9	20.5	3678.5	11.8	3659.5	23.5	3754.5	17.1	3533.3	8.6		
4	3114.8	19.9	3334.9	30.4	3660.5	20.1	3641.5	23.6	3736.5	18.6	3515.3	11.4		
5	3096.8	24.5	3316.9	19.1	3642.5	22.8	3623.5	16.2	3718.5	12.8	3497.3	15.9		
6	3078.8	20.6	3298.9	11.0	3624.5	21.8	3605.5	9.0	3700.5	9.2	3479.3	18.7		
7	3060.8	–	3280.9	9.0	3606.5	11.1	3587.5	7.7	3682.5	–	3461.3	22.8		
8					3588.5	6.9					3443.3	13.2		
9					3570.5	5.4					3425.3	9.4		
10											3407.3	–		

*In the case of AMV6, the masses are corresponding to the peptide AMV6 without the C-terminal part ESGKCTWV.*

#### Antimicrobial Activity Test

**FIGURE 5:**
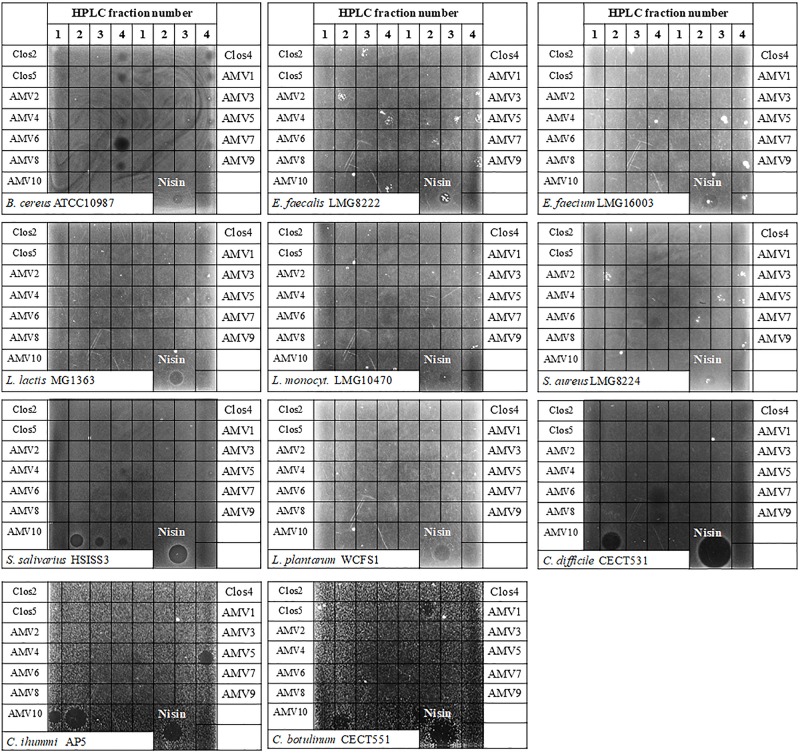
Spot-overlay test of HPLC purified fractions against Gram-positive indicator strains.

One milliliter fractions of the HPLC was collected between minutes 15 and 25. These fractions were lyophilized and stored. Because the state of dehydration is essential for the activity, and the activity of the peptides against different bacteria could be related to the state of dehydration, the four fractions corresponding to the main peaks of each lantibiotic were resuspended in 1 mL of solvent A and were assayed against Gram-positive bacteria using spot overlay test (5 μL drops). In Figure 3, the antimicrobial activity is represented. NisP was previously added into the plates for the leader cleavage. No activity was observed for the peptides Clos2, Clos4, and Clos5 against the tested bacteria, except for *B. cereus* ATCC10987 for which a small halo was observed for the fraction 4 of each peptide. Finally, the same test was performed against three strains of *Clostridium* (Figure 5), but unfortunately, no antimicrobial activity was observed.

### Chimeric Peptide Design, Expression, and Characterization

Since the specificity toward *Clostridium* could be related to the lipid II binding domain or with the other rings, and because of the relatively high activity of nisin against *Clostridium*, we decided to design new lantibiotics by a synthetic biology approach combining different parts of the putative lantibiotics and nisin.

#### Peptide Design

**FIGURE 6:**
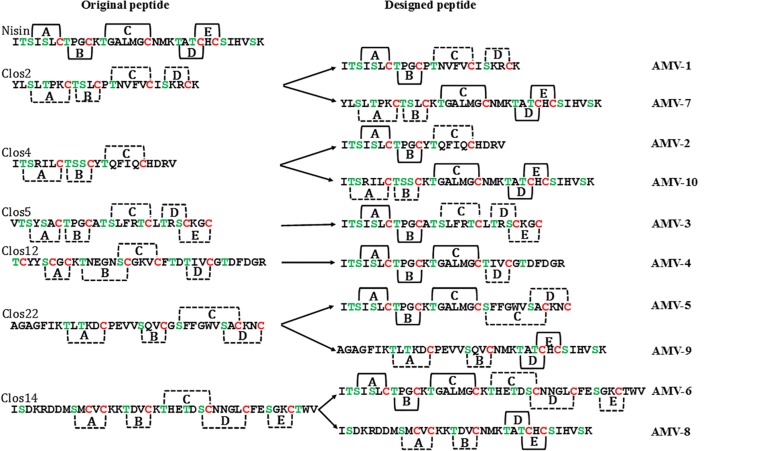
Putative structure of the designed AMV_x_ peptides. Solid line, well-known rings formation and dot line, putative ring formation.

Ten different peptides were designed (Table 5 and Figure 6). Five peptides were designed based on the peptides Clos2, Clos4, and Clos5 and the other five based on the other lantibiotics identified after the mining. Originality and new lantibiotic structure design were also considered. Depending on the nisin part used in the fusion, three kinds of peptides were designed. The first group contains those peptides for which the first two rings of nisin were fused to the last part of the putative lantibiotics. AMV1 was designed in this line by the fusion of nisin AB rings and the putative last two rings of Clos2, AMV2 with the putative last ring of Clos4 and AMV3 with the putative last three rings of Clos5. The idea, in this case, was to design the new peptides with the structure close to the wild putative lantibiotic peptide (Figure 6).

**TABLE 5:** Sequence of the nisin hybrid designed peptides.

**Peptides**	**Sequence**	**S + T**
Nisin	ITSISLCTPGCKTGALMGCNMKTATCHCSIHVSK	9
AMV1	ITSISLCTPGCPTNVFVCISKRCK	7
AMV2	ITSISLCTPGCYTQFIQCHDRV	5
AMV3	ITSISLCTPGCATSLFRTCLTRSCKGC	9
AMV4	ITSISLCTPGCKTGALMGCTIVCGTDFDGR	7
AMV5	ITSISLCTPGCKTGALMGCSFFGWVSACKNC	7
AMV6	ITSISLCTPGCKTGALMGCKTHETDSCNNGLCFESGKCTWV	10
AMV7	YLSLTPKCTSLCKTGALMGCNMKTATCHCSIHVSK	9
AMV8	ISDKRDDMSMCVCKKTDVCNMKTATCHCSIHVSK	7
AMV9	AGAGFIKTLTKDCPEVVSQVCNMKTATCHCSIHVSK	7
AMV10	ITSRILCTSSCKTGALMGCNMKTATCHCSIHVSK	10

*In shading the nisin part of the peptides. S and T positions are indicated in green while C positions are in red.*

Another group of chimeric peptides consisted of those for which the first three rings of nisin and the last part of the putative lantibiotics were combined. AMV4 was designed by the fusion of the nisin ABC rings and the last putative ring of Clos12, obtaining a peptide with four putative rings and a long C terminal tail. AMV5, obtained by the fusion with the last putative two rings of Clos22, obtaining a peptide with five putative rings (as nisin) and with a putative end ring (as described for many other lantibiotics). AMV6 was obtained by the fusion with the last three putative rings of Clos14, obtaining a long lantibiotic with six putative rings and a tail (Table 5 and Figure 6).

Finally, another group was containing peptides with the first part of *Clostridium* peptide and the last part from nisin. AMV7 and AMV10, with the first two rings of Clos2 and Clos4, respectively, and the last three rings of nisin, obtaining peptides with five putative rings closely to nisin. The peptides AMV8 and AMV9 were designed with the first putative two rings of Clos14 and Clos22, respectively, and the last two rings of nisin (with the hinge region included) (Table 5 and Figure 6).

#### AMV_x_ Cloning, Expression, and Characterization

As before, each peptide was independently cloned fused to the nisin leader and under control of the P_nis_ promoter in pNZ8048 and transformed into *L. lactis* NZ9000 pTLR-BTC strains. TCA precipitation and MALDI-ToF analysis were also performed, finding the peptides in the supernatant in all the cases, so 1 L purification was performed using the methodology described for Clos_x_ peptides.

**FIGURE 7:**
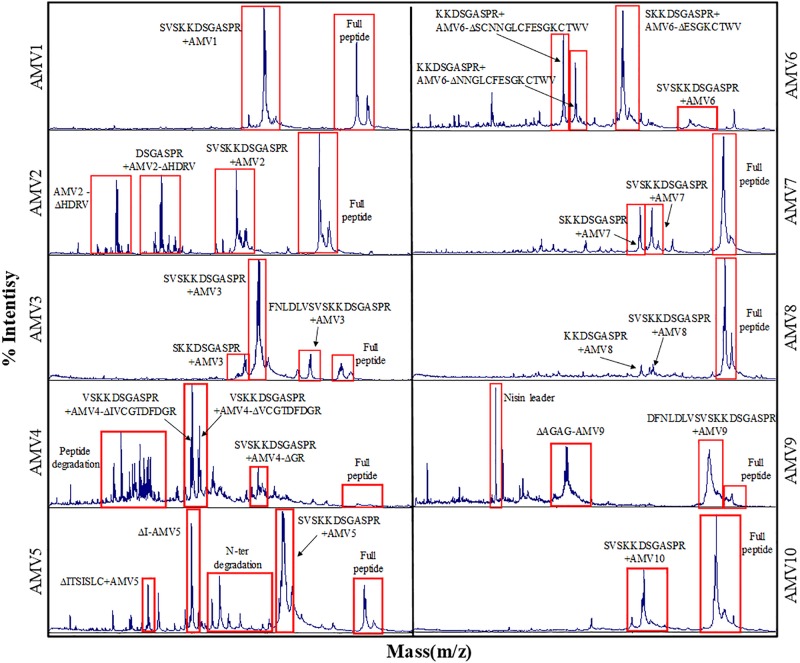
MALDI-ToF chromatogram for AMV_x_ peptides indicating different degradation levels.

The best peptide production levels were obtained for AMV5, AMV7, and AMV10, and moderate levels of production were obtained for AMV1, AMV2, and AMV3 (Figures 2B–E). In the case of AMV4, AMV6, AMV8, and AMV9, the production levels were very low (especially for AMV9) (Figure 2). As before, many peaks were observed for each peptide, suggesting different dehydration levels. As before, after MALDI-ToF analysis different degradation levels for the peptides were observed, but interestingly the N-terminal leader degradation was stronger in the case of the peptides for which the first part of nisin was used (Figure 7). This fact was especially observed in the case of the peptides AMV1, AMV2, AMV3, AMV4, AMV5, and AMV6, and to a lesser extent for the peptides AMV7, AMV8, AMV9, and AMV10 (Figure 7). In some cases, the N-terminal degradation also affected the designed peptides as in AMV5, where peaks without the first I or without the first ring (ITSILC) were observed. Interestingly, this fragment was not fully dehydrated, suggesting that the ring protects from degradation. Another example is the peptide AMV9. In this case, the complete nisin leader was observed as well as the core peptide without the first four aa (AGAG). This piece could have been released spontaneously or by a specific protease, where this sequence could be targeted. In the case of the peptides, AMV2, AMV4, and AMV6, also a putative C-terminal degradation was detected, although the peptide with higher degradation rate was AMV4. HDRV C-Ter degradation was observed for AMV2, ESGKCTWV C-Ter degradation for AMV6, and until IVCGTDFDGR in the case of AMV4.

As before, and in order to establish the dehydration level for each peptide, they were digested with NisP. The leader cleavage efficiency was also checked for these peptides, and in general, after 3 h of incubation the leader was released entirely in all the peptides with the exception of AMV5 where the leader cleavage efficiency was a bit lower (Supplementary Figure S2). The percentage of dehydration was approximately calculated considering the intensity of each peak in the MADI-ToF (Figure 4 and Table 4). Only the peptides AMV1, AMV2, AMV3, AMV6, AMV7, and AMV8 were fully dehydrated, but in general, all were dehydrated to a greater or lesser extent (Table 4).

#### AMV_x_ Antimicrobial Activity and Ring Formation

As in the case of the Clos_x_ firstly, the four HPLC fractions corresponding to the main peaks were assayed against different Gram-positive bacteria and *Clostridium*. In general, the peptides were not active against the Gram-positive bacteria assayed (Figure 5) with the exception of *B. cereus*. In this case, fraction 4 of the truncated peptide AMV6 displayed an evident antimicrobial activity, while others as AMV1 or 8 displayed a weak activity. Interestingly, the fractions 2, 3, and 4 of peptide AMV10 showed antimicrobial activity against *Streptococcus salivarius* but not against the rest of the tested bacteria (Figure 5).

In the case of *Clostridium* (Figure 5), two of the designed peptides showed an explicit activity: AMV5 and AMV10. The first one against *C. ihumii* and the other against the three *Clostridium* tested. Curiously, the fractions 1 and 2 of AMV10 were active against *C. ihumii* unlike in *S. salivarius*. A really weak activity was also observed for the fraction 2 of AMV1 and the fraction 4 of AMV6 against *C. botulinum* and *C. difficile*, respectively.

Based on these results, the peptides AMV5 and AMV10 were used for the antimicrobial test in liquid medium. The peptides were quantified using Qubit^TM^ Protein Assay Kit and a fluorimeter, and then, they were assayed by triplicate at concentration ranged from 32 to 0.007 μg/mL against six different strains of *Clostridium*. NisP was added to the culture medium to ensure the release of the leader.

According to Table 6, AMV10 displayed a broad and high antimicrobial activity against some pathogenic *Clostridia*, such as *C. difficile* or *C. botulinum* with MIC values between 0.25 and 2 μg/mL. The same activity was observed for *C. ihumii*. *C. beijerinckii* was resistant to the higher concentration used, and *C. sporogenes* was sensitive to 32 μg/mL. AMV5 was less active than AMV10 being the only sensitive bacteria *C. ihumii* and *C. botulinum* (16 μg/mL). Prenisin was used in the same conditions as a positive control. As expected, a strong activity was observed against *C. difficile*, *C. botulinum*, and *C. ihumii*. *C. sporogenes* was resistant.

**TABLE 6:** MIC values determined by broth microdilution method against several clostridial strains.

**Strains**	**MIC (μg/mL)**		
	
	**AMV5**	**AMV10**	**Nisin**
*Clostridium beijerinckii* NIZO B504	32	> 32	8
*C. botulinum* CECT551	32	2	1
*C. difficile* CECT531	> 32	2	0.03
*C. ihumii* AP5	32	1	0.5
*C. sporogenes* C22/10	> 32	32	>32
*C. tyrobutyricum* NIZO B574	> 32	> 32	4

**FIGURE 8:**
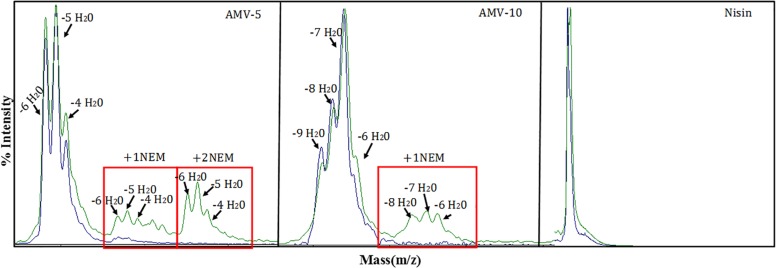
Ring formation analysis. In blue, the peptide before NEM reaction; in green, after the reaction.

Finally the ring formation was analyzed for AMV5 and AMV10 (Figure 8). In general, the ring was installed. In the case of AMV10, in some cases one of the five rings was not installed for peptides with −8, −7, or −6, but interestingly, for the −9H_2_O peptide all the ring were installed. In the case of AMV5 one ring or two rings were not installed in some cases, even in the more dehydrated peptide (−6H_2_O).

## Discussion

The treatment of infections caused by pathogenic *Clostridium* spp. poses a significant challenge in medicine, in particular by the ability of these bacteria to form spores that escape from the biocidal action of traditional antibiotics. Usually, the treatment requires the administration of antibiotics for a long time, and the broad activity of these antibiotics is related to the alteration of the gut microbiota. The most common pathogen of antibiotic-associated diarrhea is *C. difficile.* It is a Gram-positive, endospore-forming bacterium that has been recognized as one of the most frequent pathogens in nosocomial diseases, being related with a high index of morbidity and mortality (Miller et al., 2016). *C. difficile* is responsible for diarrhea and/or pseudomembranous colitis, and it is the first cause of antibiotic-associated diarrhea which is the most common adverse event related to antibiotic use (Dicks et al., 2018; Mullish and Williams, 2018). After long antibiotic treatments, the normal microbiota is damaged, and the spores of *C. difficile*, that are in virtually all healthy human, can germinate fast, releasing toxins and producing the infection. The ability of *C. difficile* spores to escape the biocidal action of the antibiotics used for its treatment produces relapses in the disease and recurrence (Mathias et al., 2019; Song and Kim, 2019).

The aim of this work was the design of new antimicrobials with potent and specific antimicrobial activity against *Clostridium* strains and especially against *C. difficile.* Starting from the premise that bacteriocins are usually active against bacteria closely related to the producing strain (Zacharof and Lovitt, 2012; Yang et al., 2014; Ventura et al., 2015), and using gene mining approaches (Weber et al., 2015; van Heel et al., 2016, 2018, 4; Montalbán-López et al., 2017), we have identified 54 putative lantibiotic sequences after the analysis of more than 560 *Clostridium* spp. genomes deposited in NCBI. From these putative lantibiotics, 10 were selected for its heterologous expression in *L. lactis* using the nisin biosynthetic machinery (Montalbán-López et al., 2017).

Among the genomes analyzed, the one from *C. ihumii* (Merhej et al., 2015) stands out since it encodes a class I lantibiotic cluster in where until 11 putative lantibiotics with a high range of diversity could be processed by a unique *lan*BTC system (Figure 1). This kind of lantibiotic island has been not described to date in other bacteria. However, it has been described that some bacteria have the ability to produce many different lanthipeptides (with the codifying gene dispersed in the genome) using only one biosynthetic enzyme as the ubiquitous marine cyanobacteria *Prochlorococcus* or *Synechococcus* (Cubillos-Ruiz et al., 2017). None of the *C. ihumii* putative peptides selected for heterologous expression was produced by *L. lactis*. In fact, only the peptides named Clos2, Clos4, and Clos5 from *C. beijerinckii* HUN142 were produced. Five different putative lantibiotics were identified in the genome of this bacteria divided into two complete clusters (one close to the other). Together with Clos2, Clos4, and Clos5, a variant of streptin was also present in the genome.

All these Clos_x_ peptides showed a broad range of hetero-dehydration and some peaks observed in MALDI-ToF were matching with N-terminal leader peptide degradation (Figure 3). The purification of the pure form of a lantibiotic when it is hetero-dehydrated is laborious because of the impossibility to completely separate one fraction from the other. However, due to the differential hydrophobic properties of the lantibiotics depending on the dehydration level, in an RP-HPLC the more dehydrated forms elute later than, the less dehydrated (van Heel et al., 2016). Based on this and because the antimicrobial activity of the lantibiotics toward specific sensitive bacteria could be dehydration-level dependent, four HPLC fractions were assayed. In general, none displayed antimicrobial activity against the Gram-positive bacteria assayed and/or against *Clostridium* strains except a weak activity of fraction 4 of each peptide against *B. cereus*.

Nisin is well known to display potent antimicrobial activity against clostridial strains. However, as other antimicrobials, nisin is not active against spores (Mazzotta et al., 1997; Le Lay et al., 2016; El Jaam et al., 2017), and although there are still studies, nisin also seems to modify the gut microbiota (Gough et al., 2018; Jia et al., 2018). Based on this and following a synthetic biology approach, 10 differences peptides (AMV_x_) were designed by a combination of parts of nisin with parts of these putative lantibiotics. The idea was to obtain peptides as active as nisin and with specificity against *Clostridium.* Similar approaches to improve the activity of lantibiotics have recently published by Schmitt et al. (2019) but unlike that work, in this case, the lantibiotic used in the hybrid peptides are putative, the rings AB(C) part of nisin were changed in some of them and the design is focused toward a specific bacterial group, i.e., Clostridia.

Two groups of peptides were designed based on the use of the rings AB/ABC of CDE/DE of the nisin in combination with the putative rings of the peptides. Unlike Clos_x_ peptides, all the new designed were heterologously expressed in *L. lactis*, but only AMV5, AMV7, and AMV10 were produced in concentrations similar to nisin production (Figure 2). After MALDI-ToF analysis, a putative N-terminal leader degradation was observed in all the peptides as well as a putative C-terminal degradation in the case of the peptides AMV2, AMV4, and AMV6 (Figure 7). Interestingly, in these peptides and according to their sequence no C-terminal ring could be formed (unlike AMV1, AMV3, AMV5) indicating a protective effect. No C-terminal degradation was observed for the peptides in which the last part of nisin was cloned (AMV7, AMV8, AMV9, and AMV10) (Figures 6, 7). Finally, the N-terminal degradation of the leader was stronger for the peptides in which the C-terminal part of nisin was not present, suggesting that this part of nisin could be implicated in the resistance to proteases of prenisin. The presence and flexibility of the hinge region could be related to a major resistance to N-terminal degradation (Yuan et al., 2004; Zhou et al., 2015). In the case of AMV9, masses corresponding to the size of the peptide without the first four aa (AGAG) as well as a mass corresponding to the nisin leader were observed after the purification. We suggest that this peptide could be cleaved by another protease from the cell, e.g., by the intermembrane CAAX (CPBP family) proteases (Pei and Grishin, 2001; Pei et al., 2011). These proteases are related (at least) with the cleavage of class IIb bacteriocins (GG, GA, AG motifs) (Oliveira et al., 2017) and after a genome screening, at least five of these enzymes (llmg_0149, llmg_0198, llmg_0736, llmg_2326, and llmg_0852) are annotated in the chromosome of *L. lactis* MG1363 (mother strain of NZ9000).

With regard to the activity, none of the designed peptides displayed antimicrobial activity against the Gram-positive bacteria tested, with some exceptions. The HPLC fraction 4 of the truncated peptide AMV6 against *B. cereus* and the fractions 2, 3, and 4 of AMV10 against *S. salivarius.* A weak activity of the fraction of AMV1 and AMV8 was also observed against *B. cereus.* Interestingly, two peptides showed an evident activity against *Clostridium* strains, the fraction 4 of AMV5 and especially the fraction 2 of AMV10. In the case of AMV10 and unlike in *S. salivarius*, the active HPLC fractions were the numbers 1 and 2. This suggests that the dehydration level could be related to the specificity against certain bacteria.

Finally, the peptides AMV5 and AMV10 were selected for an extensive test against six different *Clostridium* strains. Concentrations ranging from 32 to 0.007 μg/mL were tested. We found that AMV10 is a potent peptide against pathogenic clostridia such as *C. difficile* or *C. botulinum*. Notably, AMV10 was not active against *C. beijerinckii*. AMV10 was obtained by the combination of the first two putative rings of Clos4 with the rings CDE of nisin, and Clos4 is a putative lantibiotic identified in the genome of other *C. beijerinckii* (Figure 6). This suggests that these strains could present specific resistance to these peptides and that the specificity toward clostridia is more related to the first rings (i.e., lipid II binding domain) than with the last one. However, *C. beijerinckii* was sensitive to AMV5 at the higher tested concentration as well as *C. ihumii* and *C. botulinum*. In this case, the first rings of the designed peptides are from *C. perfringens*. The rest of the species tested were resistant (Table 6). Respect the ring installation, only the peptide AMV10 with −9H_2_O is able to form all the rings. That means that probably only around the 10% of this peptide (Table 4) is the most active.

## Conclusion

The primary objective of this work was the development of new antimicrobials with high specificity and activity against specific clostridial strains, especially pathogenic *Clostridia* as *C. difficile.* Using the combination of two different methodologies, genome mining of clostridial genomes and synthetic biology, 20 different peptides were heterologously expressed in *L. lactis* obtaining at the end two peptides (AMV5 and specially AMV10) that fulfilled the requirements set in this work: good heterologous expression levels and high specificity and activity toward *Clostridium.* The high specificity and activity observed for peptide AMV10 makes it a good candidate as an alternative to traditional antibiotics in the treatment of *C. difficile* infections, avoiding the side effects and the damage on the gut microbiota.

Finally, the methodology applied in this work has shown its robustness in the identification and design of new peptides with specificity and high activity against specific bacteria and could be applied for other fastidious bacteria which are hard to treat, such as *Mycobacterium* or Gram-negative/positive ESKAPE bacteria.

## Data Availability

The datasets generated for this study are available on request to the corresponding author.

## Author Contributions

OK supervised the work. RC and OK conceived and designed the experiments. RC, AM-V, and AJ collected and analyzed the data. RC and OK wrote and reviewed the manuscript. All authors read and approved the final manuscript.

## Conflict of Interest Statement

The authors declare that the research was conducted in the absence of any commercial or financial relationships that could be construed as a potential conflict of interest.

## Supplementary Material

FIGURE S1 MALDI-ToF data of the NisP leader cleavage efficiency for Clos2, Clos4, Clos5, and nisin during 3 h.

FIGURE S2 MALDI-ToF data of the NisP leader cleavage efficiency for AMV_x_ peptides before (in blue) and after 3 h (in green).

TABLE S1 Putative lantibiotics identified in *Clostridium* ssp. genomes after the genome mining, and NCBI genome reference number.
