# Supplementary figures and images for: Design and Expression of Specific Hybrid Lantibiotics Active Against Pathogenic Clostridium spp
Source: Front Microbiol. 2019 Sep 24;10:2154. doi: 10.3389/fmicb.2019.02154 (PMC6768957; doi:10.3389/fmicb.2019.02154)

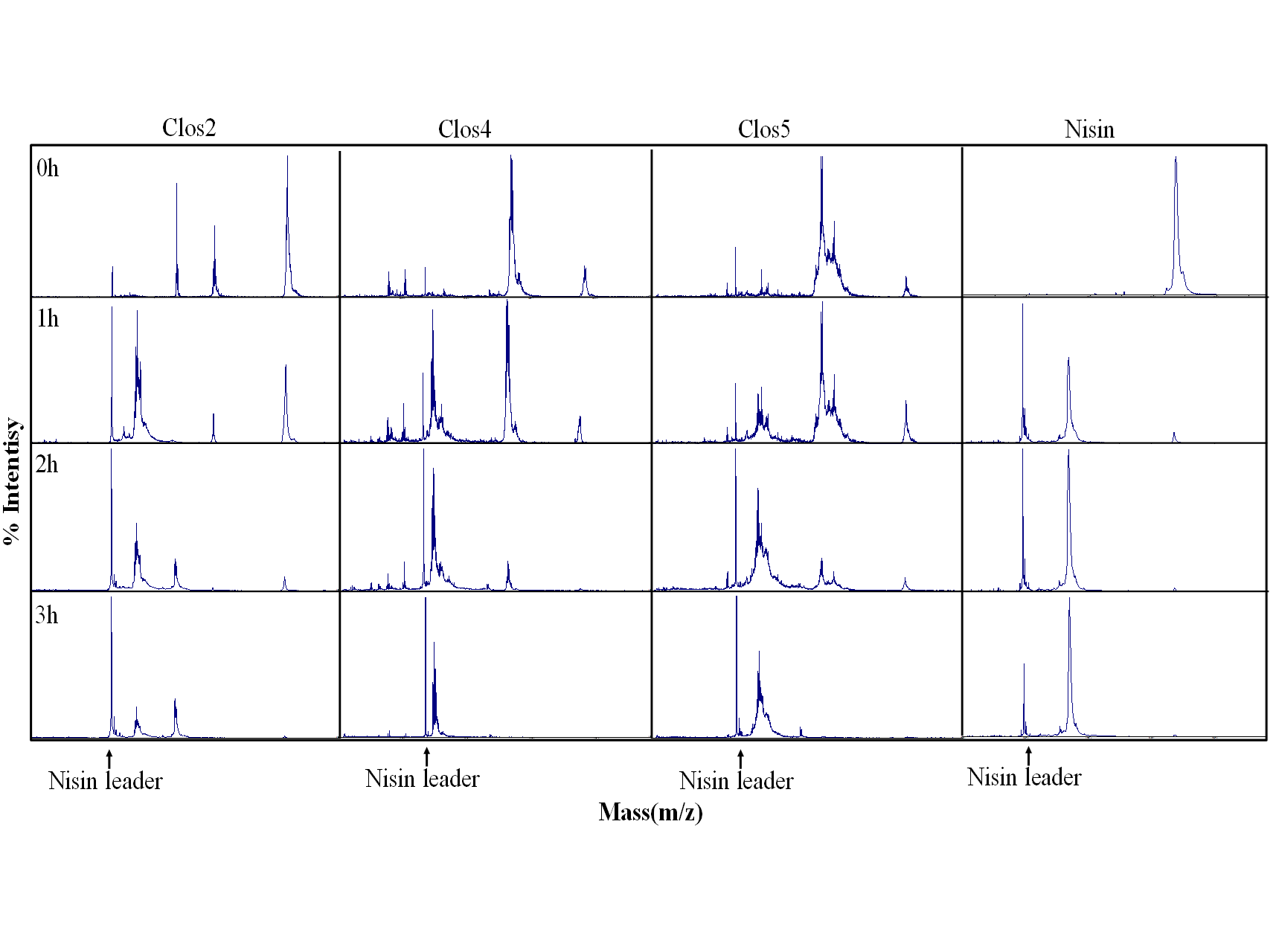

Supplement: FIGURE S1 — MALDI-ToF data of the NisP leader cleavage efficiency for Clos2, Clos4, Clos5, and nisin during 3 h. [file Image_1.TIF]

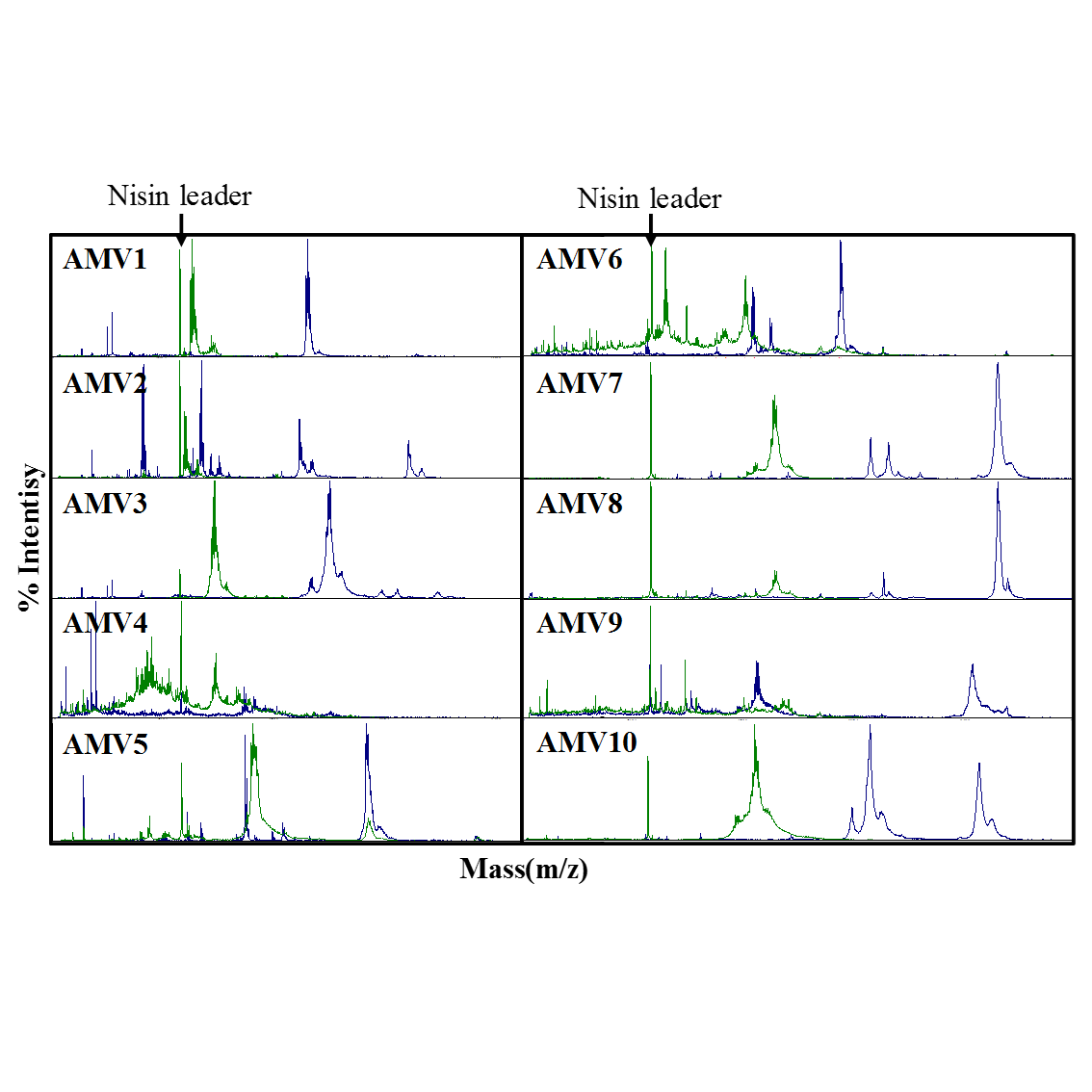

Supplement: FIGURE S2 — MALDI-ToF data of the NisP leader cleavage efficiency for AMVx peptides before (in blue) and after 3 h (in green). [file Image_2.TIF]
