# Supplementary material for: Design and Expression of Specific Hybrid Lantibiotics Active Against Pathogenic Clostridium spp
Source: Front Microbiol. 2019 Sep 24;10:2154. doi: 10.3389/fmicb.2019.02154 (PMC6768957; doi:10.3389/fmicb.2019.02154)
Supplement: TABLE S1 — Putative lantibiotics identified in Clostridium ssp. genomes after the genome mining, and NCBI genome reference number. [file Table_1.DOCX]

| **Origin** | **Putative lantibiotic sequence** | **NCBI reference genome sequence** |
| --- | --- | --- |
| *C. beijerinckii* HUN142 | MIK**LDDFDL**KIKKDDNKTGKVTPQVNSRYACTPGSCWKWVCFTTTAK | NZ_JHXK01000032 |
|  | VGK**LDDFDL**DVKVKINSKKGIKPSYLSLTPKCTSLCPTNVFVCISKRCK |  |
|  | MAKLG**DFDLDL**KVKIKPKGGVTPATVSRFNCTLFGCIKVKDNI |  |
|  | MGK**LDDFDLD**VKVKATPKGGVKPSITSRILCTSSCYTQFIQCHDRV |  |
|  | MGK**LDNFDLD**VKIKKDEKRGVKPSVTSYSACTPGCATSLFRTCLTRSCKGC |  |
| *C. botulinum* H04402 065 | MKNNEVCKNAGFISEDELVELVDNSDIS**GG**TAASAAAVSATVASATAVSALFTVTSACTTKCK | NC_017299 |
|  | MIKNPIKRQSEDVKLPCGDTKVEITENQGLDVT**GG**TFSEGTISITLSVYMGNDGKVCTWTVECQNNCSHKK |  |
|  | MYFNFYRVVFGFLCYLHCIDIEKDYIYIIKSQK |  |
| *C. botulinum* strain CDC41370 | MKNNEICKNAGFISEDELVELVDNNDIS**GG**TASAISATVASATAVSALFTVTSACTKKCK | NZ_LAGI01000027 |
|  | MIKNPIKRQSEDVKLPCGDTKVEITENQGLDVT**GG**TFSEGTISITLSVYMGNDGKVCTWTVECQNNCSHKK |  |
|  | MRYRYIQGEDKFIYMLSIIDALDRSIIDYHMKFRCESEDVIELTDKGLIGRGWCNKTYYKN |  |
|  | LDILCLRLFIDISLALQAVQTPTEALILHSDLEYQHTSSSFKEYINRVLN |  |
|  | MSNFNE**FELDL**QNEKIQNEAASERVKFTTWDCVASSIFNCPTLKCPTKGVLVCPQPPKPVNTKSQCSSTASCRTTFKK |  |
|  | MEDKAQFGGHRGAASPKLQRKVPQSTAKYRKVGHSEWYNEYSDTPWHQAERVKTVQLVLGMPKKQIFCKI |  |
|  | MGKMD**DFDLDL**RKIAENGNSANALSASDMITSEIISKVTETITRTFKGQCVSVETPTTGMTSACCKKGGTDVEPQCVP |  |
| *C. cellulovorans* 743B | MVSFCTAAFPWVAFSIGLAVFLTYTNSKTKLKKMENKNF | NC_014393 |
|  | MLCSVWKFFLDAFYHKLSSMSISGLYFFYVIIYIE |  |
|  | MTNYKIGQTFEQKNYEEMASCQVAGDGFIFTTNQITVSYCPTLTIPHIPTITTPKLTIQ |  |
|  | MANYKIGAIFEQKNYEEMASSQMT**GG**DGFVTVTSPQYTLSCCITWTIPSIKLTV |  |
|  | MQNYESKAGFISEMELDELVSNKTV**GG**ATTVPCAIAIIGITLSAGICPTSACSKDCPWNN |  |
|  | MKNYEELFNEVNENASLQAELN**GG**SIATTIVCTIAQSLLGCVGSYVLGNKGYGCTVTNECMSNCR |  |
|  | MGSLKKITLE**DLDLDF**QIGDNNEEFLYL**GG**DSKGKLDLVGSPSVINSSLNFIQFIKTNRPVTKYTYSERGCC |  |
|  | MGDLKKLNLS**DLDLD**MQVGEINEEFINVSGEGKGDYSGSAASIAYSMMTLGQYWKGDTSTAKYTYSERGCC |  |
| *C. hylemonae* DSM 15053 | MQREEKNVEITGDLSLEFKEMQKLVDEEVGVPYSTWSKACTTFFTIICC | NZ_GG657760 |
|  | IIQTILSVCGGISII**GG**TGAIVWKCCGRRSKLPSE |  |
| *C. ihumii* AP5 | MPNYKE**FDLDI**RNEKNNLKSMNSKKRSDGGTCYYSCGCKTNEGNSCGKVCFTDTIVCGTDFDGR | NZ_CCAT010000042 |
|  | MPNYKD**FDLDL**QNNKLSKKYESVNRGNKTYNRDCGYKTHEPDSCGNSCFTSAKCDWA |  |
|  | MPNYKD**FDLDI**RNVRFQGRIDDRPGRTSIEVKEQSICIACKPKTGGSGSGGGSKTHEDSAW |  |
|  | MPNYKD**FDLDI**QNIKMNKINDKRRYPISDKRDDMSMCVCKKTDVCKTHETDSCNNGLCFESGKCTWV |  |
|  | MPNYKD**FDLDI**QNSKLGVDSSRKVLPPTFSYEYDKLSECRCRPKTQTCATHCSCATYCNGSCNQHTDCAL |  |
|  | MPNYKE**FDLDI**RNSKNGINMYGPSAVIVPATDGGGKKTVCGRTCNGSACNPNSCQTRCIKPAD |  |
|  | MPNYKD**FDLDI**QNNKSSVNSIKTTTMPPTFSYEYDQYSECVCKPKTRNSCVTYCNGSCNQHTDCTL |  |
|  | MPNYKD**FDLDL**RNNTVKGNNSKNENIQGKKKTDGCYTYGNRSCPNTMCAY |  |
|  | MPNYKD**FDLDI**QNNKGSSKTSLGKELSNTGNYYDPLSECRCKPKTYTKRLKTCNVKECMY |  |
|  | MPKYND**FNLDI**QTDNKNCHTTKLTIEVKHKENKGGNMATWSTHCY |  |
|  | MPNYKD**FDLDL**TNKKVITNEIKDKTIQANIARTYGPVKSCQYICRLSENIEDCQ |  |
| *C. josui* JCM 17888 | MCGPWELYLKYTFLSSIFPHSFLAVCLLSDESGGVDKIGVSISIVE | NZ_JAGE01000001 |
| *C. perfringens* B ATCC 3626 | MSEIDSKKIVGDTFEDMSIWEMTMVQGSGDMEPNSLTVASAVLMSAAATGSFSIAVTKTVKGKC | NZ_ABDV01000024 |
|  | MSEINMKKIVGDTFEDMSIAEMTLVQGSGDVNGEVTTSPACVYVSVAASRASSQKCGQAAGAIASFVSTAVLSAVKC |  |
| *C. perfringens* CPE str. F4969 | MMKQLDKKSKTGIYVQVASDKELELLV**GG**AGAGFIKTLTKDCPEVVSQVCGSFFGWVSACKNC | NC_007772 |
| *C. perfringens* D JGS1721 | MMKQLDKKSKTGIYVQVASDKELELLV**GG**AGAGFIKTLTKDCPEVVSQVCGSFFGWVSACKNC | NZ_ABOO01000006 |
|  | LFYKVLLLLGWRDKMKLIRIISGVVSIFFIGCAAYGYYSSKTLYLADVILGLIAISVFAFSFLKNSKNN |  |
| *C. senegalense* JC122 | MSNYND**FDLDL**KMVSENGAQSKGVSDYTVDIITSALTCWVKISKALNCTNGRECAMPTKDRPAASCHRAMAGAVQARC | NZ_HE611055 |
| *C. sordellii* W10 | MSNFNE**FELDL**QNEKIQNEAASERVKFTTWDCVASSIFNCPTLKCPTKGVLVCPQPPKPVNTKSQCSSTASCRTTFKK | NZ_CDNY01000001 |
| *Clostridium* sp. ASF502 | MRTACTRRSSTTGRICARNPCARDRAGIRKRHGVTAQAGSRCQE | NZ_KB822463 |
|  | MSTSLYQNLIQTANQFCNQYPSCPYDSCSIK |  |
| *Clostridium* sp. BNL1100 | MTSLLSPLKISIVLNPTFYTIILRKANGIKNIRKLCWTSAIHINPKTSSTNN | NC_016791 |
|  | LVLWTVPKVCC**GG**KLIGYNLRGLFPDVNKACFHK |  |
| *Clostridium* sp. KLE 1755 | MGKMDD**FDLDL**RKIAENGNSANALSASDMITSEIISKVTETITRTFKGQCVSVETPTTGMTSACCKKGGTDVEPQCVP | NZ_KE992698 |
|  | LLAKTRKTENNLFSNHNIEGIKLCQREICYTAPKRYGGEVCESD |  |
| *Clostridium* sp. BR31 | MDD**FDLDL**RKIAENGNSANALSASDMITSEIISKVTETITRTFKGQCVSVETPTTGMTSACCKKGGTDVEPQCVP | NZ_MIEH01000017 |
| *Clostridium* sp. KNHs205 | MEVKEMTTKVTRVKTGNQFNHPAGDIPAEISEIVSLRESKNPDAIYTITVGCSGFLTLICC | NZ_JNLE01000003 |
| *C.* saccharobutylicum DSM 13864 | MRETLLVVLMLLFVILAAAMIFPMPPIIYGAIMSLIILCIIIIISIYILIIKKNH | NC_022571 |

**Supplementary Table 1:** Putative lantibiotics identified in *Clostridium* ssp. genomes after the genome mining, and NCBI genome reference number. In red and/or green some lantibiotics related domains.
